# Comprehensive Exploitation of Time- and Frequency-Domain Information for Bearing Fault Diagnosis on Imbalanced Datasets via Adaptive Wavelet-like Transform General Adversarial Network and Ensemble Learning

**DOI:** 10.3390/s25072328

**Published:** 2025-04-06

**Authors:** Huachao Jiao, Wenlei Sun, Hongwei Wang, Xiaojing Wan

**Affiliations:** Intelligent Manufacturing Modern Industry College, Xinjiang University, Urumqi 830049, China; jhc_xj@163.com (H.J.); wanghongwei_xju@126.com (H.W.); wanwan1027@126.com (X.W.)

**Keywords:** adaptive wavelet-like transform, GAN, bearing fault diagnosis, imbalance data, ensemble learning

## Abstract

The vibration signals of faulty bearings contain rich feature information in both the time and frequency domains. Effectively leveraging this information is crucial, especially when addressing imbalanced bearing fault datasets, as it can significantly enhance the performance of fault diagnosis models. However, existing GAN models and diagnostic methods do not fully exploit these domain-specific features. To overcome this limitation, a novel fault diagnosis method is proposed, based on the Adaptive Wavelet-Like Transform Generative Adversarial Network (AWLT-GAN) and ensemble learning. In the first stage, AWLT-GAN is used to balance the bearing fault dataset by integrating time- and frequency-domain feature information. AWLT-GAN embeds an adaptive wavelet-like transform neural network into the generator as an adaptive layer and employs a dual-discriminator architecture. This design allows the network to simultaneously learn fault characteristics from both domains within a single training session, enhancing the quality of the synthetic fault data. Next, an ensemble learning approach is applied, combining time- and frequency-domain models, with the final classification determined through a soft voting mechanism. Experimental results demonstrate that the vibration signals generated by AWLT-GAN effectively replicate the feature distribution of real data, confirming its high performance. The fault diagnosis model, developed using these high-quality synthetic samples, accurately captures fault characteristics embedded in both the time and frequency domains, resulting in enhanced diagnostic performance. The proposed approach not only addresses the imbalance in bearing fault datasets but also significantly improves diagnostic accuracy.

## 1. Introduction

Rotary mechanisms are extensively utilized in a variety of functional machinery across industries such as agriculture, wind power, and transportation, where they play a crucial role in transmitting motion and converting torque. Bearings, as a vital component of these rotary mechanisms, have a direct impact on the efficient and stable operation of functional machinery. The real-time acquisition of bearing health status and the formulation of maintenance strategies in accordance with this status can effectively enhance the overall economy, safety, and stability of the machinery [1].

Given the importance of bearing health assessment, various signal processing and diagnostic approaches have been developed over the years, including both traditional techniques based on time-domain or frequency-domain analysis and modern machine learning-based methods [2]. Although traditional time-domain and frequency-domain signal processing methods (e.g., FFT-based spectral analysis) have been widely used in bearing fault diagnosis, these approaches rely heavily on handcrafted feature engineering and expert knowledge. Furthermore, they often struggle to capture complex, nonlinear fault patterns, especially under varying operational conditions or in noisy environments. In contrast, machine learning-based models, especially deep learning techniques, offer superior performance by automatically learning hierarchical features directly from raw or preprocessed data. This allows them to generalize better across different fault types and operating conditions [3]. Deep learning-based fault diagnosis models are a vital means of perceiving bearing health. With the advancement of related software and hardware, the deployment of these models has become increasingly convenient, highlighting their efficiency and accuracy. Consequently, they are being widely applied in various fields for bearing fault diagnosis [4,5,6]. The construction of deep learning-based fault diagnosis models relies on sufficiently large and balanced datasets. However, in industrial practice, bearings mostly operate normally, and faults, when they occur, typically affect machinery operation and are promptly addressed. The timing and location of bearing faults are influenced by surrounding components and loads, making them difficult to predict. This results in a scarcity of high-quality bearing fault samples and an imbalance in the quantity of different fault samples, leading to an imbalanced bearing fault dataset.

In recent years, GANs have been increasingly used to address the issue of imbalanced bearing fault data [7,8]. The reason for this is that GANs, through adversarial learning, can effectively learn the characteristics of real data and generate new data that closely resemble the real data [9,10]. Huang et al. [11] enhanced their generative model by incorporating a data autoencoder at the input stage and optimizing the discriminator’s loss function, thereby generating one-dimensional signals with a probability distribution closely resembling real data, exhibiting heightened resilience to label noise and efficaciously supporting fault diagnosis in wind turbine gearbox bearings. Li et al. [12] leveraged the GRU network’s prowess in time-series data handling, integrating a positional attention mechanism and a calibration technique for fault pulse probability distributions, thereby crafting a GAN generator that meticulously preserves temporal fault data integrity, yielding more precise and realistic fault signal synthesis. Luo et al. [13] proposed the Enhanced Relative Generative Adversarial Network, a model featuring one-dimensional convolutional layers and spectral normalization layers to reconstruct its generator and discriminator, thereby improving the adaptability of network parameters to gradient penalty calculations, enhancing the ability to capture fault features, and reducing sensitivity to data quantity, allowing the generation of high-quality one-dimensional vibration signals to address the issue of imbalanced bearing fault data. Liu et al. [14] utilized optimized CNNs within the GAN model to better learn data features and generate higher-quality one-dimensional vibration signals, thereby addressing the issue of data imbalance. Luo et al. [15] proposed the DWGANGP model, which is designed to generate one-dimensional vibration signals that exhibit characteristics similar to the original samples.

The aforementioned literature employs the adversarial structure of GANs to learn features from the time domain of bearing fault vibration signals, generating fault data whose time-domain feature distribution closely resembles that of real samples, thereby addressing data imbalance issues. Simultaneously, the frequency domain of bearings also contains abundant fault information. Many scholars utilize GAN methods to generate data with similar frequency domain distributions, balancing the fault dataset and thus constructing fault diagnosis models. Zhang et al. [16] proposed a Convolutional Block Attention Mechanism Conditional Regularized Least Squares Generative Adversarial Network (CBAM-CRLSGAN), which learns genuine fault features from frequency-domain samples procured via Fourier transform, thereby generating high-quality fault data utilized to establish a diagnostic model. Wang et al. [17] proposed a deep convolutional conditional generative adversarial network (DCCGAN) model based on a CNN, which learns the feature distribution of real data from frequency-domain data obtained through Fourier transform, generating high-quality fault samples to address the issue of imbalanced data in rotating machinery fault diagnosis. Tao et al. [18] utilize short-time Fourier transform (STFT) to convert the original 1-D vibration signals into 2-D time–frequency maps, and then train a CatGAN model to generate fake samples with a similar distribution to the real time–frequency maps. The ST-CatGAN method can effectively identify mixed fault conditions without manual labeling and demonstrates strong robustness. Wang et al. [19] propose a semisupervised auxiliary classifier generative adversarial network (SACGAN) that also utilizes the STFT to convert one-dimensional time-domain vibration signals of bearings into two-dimensional time–frequency images, which serve as the input for the SACGAN to generate high-quality multi-mode fault samples. Liu et al. [20] proposed the condition multidomain generative adversarial network (CMDGAN), which employs a self-adaptive feature extraction module to generate sample labels, utilizes binary discriminators for supervised adversarial training, and integrates time-domain and frequency-domain information to effectively capture the distribution features of limited original samples, resulting in generated samples that resemble the original samples in both the time and frequency domains. Liu et al. [21] propose an improved multi-scale residual generative adversarial network (MsR-GAN), which extracts two-dimensional time–frequency features from the original vibration signals using frequency slice wavelet transform (FSWT) to generate high-quality time–frequency features for balancing the fault data distribution, and finally constructs a fault diagnosis model based on the balanced data. Zhao et al. [22] employed an autoencoder to evaluate the similarity and filter the generated 2D images, which are obtained through the wavelet transform, thereby enhancing the diversity of GAN-generated samples and producing high-quality time–frequency samples that can effectively mitigate the issue of data imbalance in mechanical system fault diagnosis.

**Figure 1:**
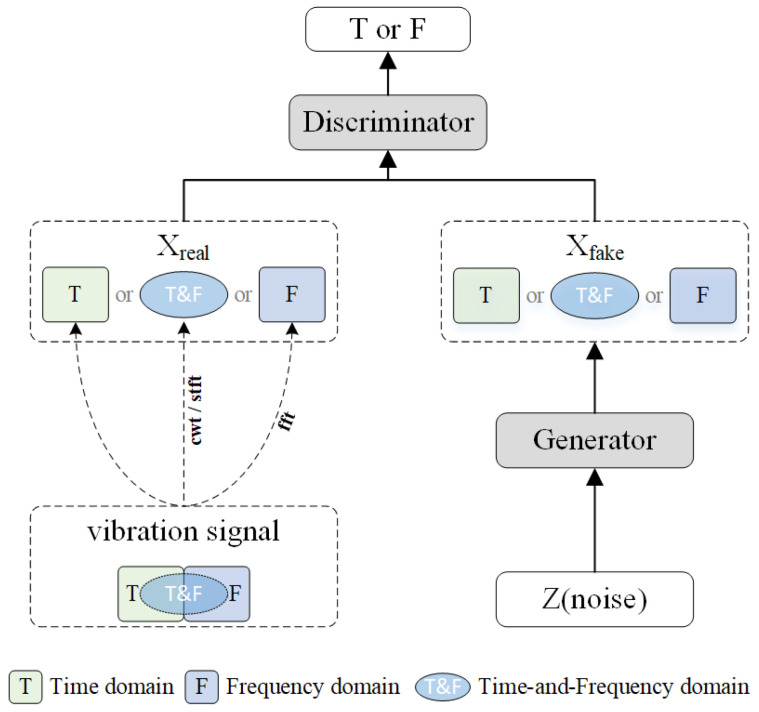
Structure of traditional GAN for bearing fault diagnosis.

As highlighted by the literature review, the main optimization focus for addressing data imbalance with GANs lies in enhancing the model’s feature extraction capabilities and improving the stability of the training process [23,24]. The goal is to enable the GAN to generate samples that are more similar to the training set at the feature level, thus helping to balance the dataset. However, there has been limited exploration of the consistency of the frequency-domain information in the one-dimensional vibration signals generated by GANs and the time-domain information in the spectral maps when compared to the actual training samples. This oversight is largely due to the original design of GANs, which aims to generate samples similar to the training set at the feature level, naturally neglecting aspects not covered in the training data. As illustrated in Figure 1, the vibration signals from faulty bearings typically contain three types of information: time-domain, frequency-domain (usually obtained through Fourier transform), and time–frequency domain (usually obtained through wavelet transform or short-time Fourier transform). However, due to the limitations of traditional GAN models, the generator can typically only utilize one type of information, such as if X_real is time-domain, making it nearly impossible for X_fake to generate frequency-domain data.

Although the time-domain and frequency-domain information in faulty bearing vibration signals contains rich fault characteristics, and using GAN networks to generate fault data can effectively address the imbalance in bearing fault data, when bearings are affected by surrounding components, resulting in a low signal-to-noise ratio, extracting comprehensive feature information from the fault data becomes crucial to significantly improve the performance of the fault diagnosis model [25,26]. If the samples used by the GAN contain only one type of information, even with an excellent feature extractor, discrepancies may still exist between the generated samples and the real training set. Consequently, the high-dimensional feature distribution extracted by a fault diagnosis model built on such a dataset may not align with the feature distribution based on real samples, potentially compromising the model’s accuracy. Therefore, to effectively mitigate the data imbalance issue in bearing fault diagnosis, the GAN model should fully utilize diverse types of data, which is a key strategy for improving the accuracy of the fault diagnosis model [27,28].

Like traditional GAN models, existing ensemble learning methods encounter comparable issues in the use of training set samples. These studies, [29,30,31,32], have employed ensemble learning approaches and incorporated feature extraction networks optimized through various methods, enabling fault diagnosis models based on ensemble learning to more effectively utilize the characteristic information embedded in the signals. However, these training set samples also use time-domain, frequency-domain, or partial time–frequency data from fault signals individually.

To fully exploit the fault feature information in both the time and frequency domains of bearing signals, and to address the accuracy issues of bearing fault diagnosis models affected by data imbalance, a novel method that integrates AWLT-GAN and ensemble learning is proposed. This method generates high-quality training samples via AWLT-GAN, enabling the ensemble learning-based fault diagnosis model to more effectively harness the fault information within the signals, thereby enhancing the precision of fault diagnosis. The main contributions of this paper can be summarized as follows:

1. AWLT-GAN combines the adaptive wavelet-like transform (AWLT) with generative adversarial networks (GANs) to enhance the learning of both time- and frequency-domain features in a single training iteration. This integration allows the model to generate synthetic fault data with higher fidelity and accuracy, particularly in imbalanced datasets. Compared to traditional GAN models, the AWLT-GAN introduces a novel dual-discriminator structure, which significantly improves the diagnostic performance by effectively capturing complex signal characteristics in both domains.
2. The adaptive wavelet-like transform neural network (AWLT) is specifically designed to overcome the limitations of conventional wavelet transforms in fault diagnosis. This network establishes a mapping between the time and frequency domains of the signal, allowing for the adaptive selection of wavelet basis functions based on the signal’s characteristics. This innovation ensures superior extraction of both time- and frequency-domain features in a single iteration, leading to more accurate and diverse fault data generation.
3. An advanced ensemble learning framework is introduced, combining 1D CNN models for time-domain feature extraction with 2D CNN models for frequency-domain analysis. This comprehensive approach leverages soft voting mechanisms to achieve more accurate fault diagnosis. By integrating both time- and frequency-domain data, the ensemble model provides superior classification performance compared to models that rely solely on one type of feature, thus improving the robustness of the diagnosis under varying operational conditions.

The main contents of this paper are as follows. Section 2 describes the basic theory. Section 3 gives the construction process of the proposed method. Section 4, Section 5 and Section 6 show the results of the experiments and the comparison of the different methods. Section 7 concludes the whole article.

## 2. Theoretical Fundament

### 2.1. Wavelet Transform

The wavelet transform was developed to address the Fourier transform’s lack of local analysis capability in the time domain when processing non-periodic or non-stationary signals. Based on the characteristics of wavelet functions, the wavelet transform can precisely analyze signals in both the time and frequency domains, offering excellent local analysis capabilities and effectively handling non-stationary signals. By adjusting the wavelet shape, the wavelet transform can have longer time intervals to obtain more low-frequency information from the signal, and shorter time intervals when more precise high-frequency information is needed [33]. The theoretical foundation is as follows.

The signal X(n) of length *N* undergoes a wavelet transform to obtain the wavelet transform coefficients $W_{a,b}$, as shown in Formula: (1).

(1) $$\left\{\begin{array}{c} W_{a,b}=\sum_{i=0}^{N-1}X\left(i\right)\psi_{a,b}\left(i\right)\\ \psi_{a,b}\left(i\right)=\frac{1}{\sqrt{a}}\psi \left(\frac{i-b}{a}\right) \end{array}\right.$$

where ψ(i) is the wavelet function, *a* is the scale factor with 0<a<M, and *b* is the translation factor with 0≤b<M.

Given $W_{a,b}$, the signal X(n) can be reconstructed using the inverse wavelet transform:

(2) $$\left\{\begin{array}{c} X\left(n\right)=\frac{1}{C_{\psi }}\sum_{i=1}^{M-1}\sum_{j=0}^{N-1}W_{i,j}\psi_{i,j}\left(n\right)\\ C_{\psi }=\sum_{j=0}^{N-1}\frac{|\hat{\psi }{\left(\omega \right)|}^{2}}{\left|\omega \right|} \end{array}\right.$$

where $C_{\psi }$ is the normalization factor of the Fourier transform $\hat{\psi }\left(\omega \right)$ of the wavelet function ψ(n) in the frequency domain, where ω represents the angular frequency.

### 2.2. GAN

In 2014, Goodfellow et al. [34] proposed the GAN network, inspired by game theory, as a data generation network based on a multi-layer perceptron structure. The network consists of a generator and a discriminator. The generator maps random data from a prior distribution (e.g., Gaussian distribution) to new data that resemble the distribution of real data through a multi-layer perceptron. The discriminator extracts features from both real and generated data to distinguish between real and generated data. During training, the generator aims to make the generated data as indistinguishable from the real data as possible, with its loss function defined as Equation (3). For the discriminator, the training objective is to efficiently differentiate between real and generated data, with its loss function defined as Equation (4). The objectives of the generator and discriminator are adversarial, resembling a game process. Thus, the training of the entire GAN network is transformed into a minimax problem of the discriminator’s recognition probability.

(3) $$L_{G}=-E_{z∼p_{g}\left(z\right)}\log\left[1-D(G(z))\right]$$

(4) $$L_{D}=E_{x∼p_{d}}\left[\log D\left(x\right)\right]+E_{z∼p_{g}}\left[\log\left(1-D\left(G\left(z\right)\right)\right)\right]$$

where *E* denotes the expected value. $p_{g}$ represents the distribution of data $G_{z}$ synthesized from noise *Z*. $p_{x}$ signifies the distribution of sample *x*. *D* symbolizes the discriminator.

## 3. Proposed Method

### 3.1. Adaptive Wavelet Transform

**Figure 2:**
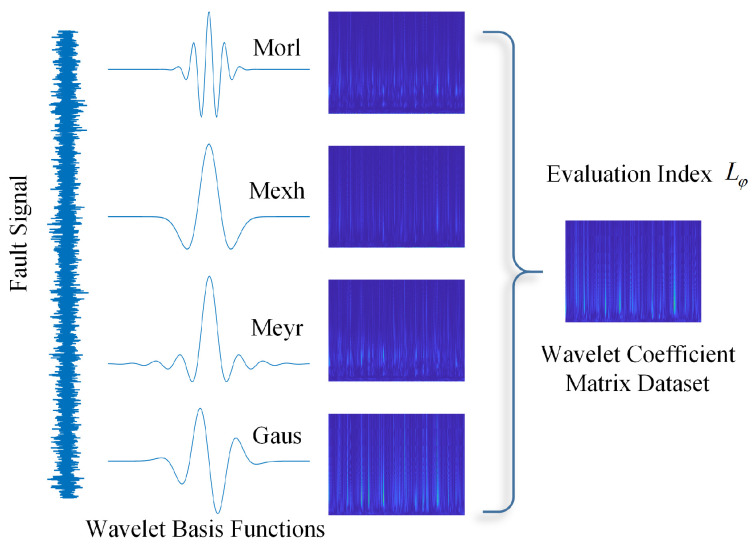
Schematic diagram of adaptive wavelet transform.

Wavelets come in various types of base functions, which provide engineers with flexibility and variability in signal analysis. For the same signal, wavelet spectra obtained using different base functions are different, and selecting an appropriate base function can highlight the feature information of interest in the signal. By providing an appropriate evaluation index for the wavelet transform results, the computer can quickly filter out suitable base functions, laying a data foundation for the construction of the wavelet neural network. To this end, an index for evaluating the quality of the wavelet transform results has been introduced. Based on this index, the wavelet basis function can be adaptively selected during the wavelet transform of the signal, yielding a wavelet coefficient matrix that best represents the fault characteristics of the signal. Subsequently, a more optimized dataset of wavelet coefficient matrices can be obtained. The specific method is illustrated in the Figure 2.

Once the fault characteristics of interest are identified, the wavelet coefficient energy can be used as an indicator of the energy of the fault characteristics in the signal. The formula is as follows:

(5) $$E_{a}\left(W\right)=\sum_{b=1}^{N}{\left(\frac{W_{a,b}-\min W_{a,b}}{\max W_{a,b}-\min W_{a,b}}\right)}^{2}$$

If the characteristic of interest is present in the signal and has a high similarity to the wavelet basis function, then after the wavelet transform, the energy indicator $E_{a}\left(W\right)$ will be greater when the scale factor is *a*. Therefore, this can be used as one of the criteria for selecting the wavelet basis function.

If the energy measurement values are the same, due to the impact characteristics of bearing faults, a suitable wavelet basis function should have more concentrated energy in the time direction, i.e., its entropy value should be low. A selection criterion based on Multi-Scale Reverse Dispersion Entropy [35] can be designed as follows:

(6) $$MRDE\left(W\right)=\sum_{i=1}^{c^{m}}{\left(P\left(\pi_{i}\right)-\frac{1}{c^{m}}\right)}^{2}$$

where *c* is the number of classes, *m* is the embedding dimension, π is a dispersion pattern related to W, and P(·) is the probabilities for each dispersion pattern. The specific settings can be referred to in [35,36,37].

A suitable wavelet basis function should maximize $E_{a}\left(W\right)$ and $MRDE_{a}\left(W\right)$. Therefore, the objective function for selecting the wavelet basis function is

(7) $$L_{\psi }=\underset{\psi \in A}{\mathrm{argmax}}\left(E_{a}\left(W\right)\cdot MRDE_{a}\left(W\right)\right)$$

where ψ is the wavelet basis function and *A* is the set of candidate wavelet basis functions. Based on the literature review, the candidate wavelet basis functions in this study are as follows: Morlet wavelet, Mexican Hat wavelet, Meyer wavelet, and Gaussian wavelet.

The joint optimization of $E_{a}\left(W\right)\cdot MRDE\left(W\right)$ (Equation (7)) ensures the selected basis simultaneously maximizes fault signature prominence in both time and frequency domains while avoiding artifacts from predefined basis mismatch (e.g., Morlet’s oscillations may dilute transient impacts). The implementation involves three steps: 1. Compute wavelet coefficients $W_{a,b}$ for all candidate bases ψ∈{Morlet,MexicanHat,Meyer,Gaussian}. 2. Calculate $L_{\psi }=E_{a}\left(W\right)\cdot MRDE\left(W\right)$ across scales *a*. 3. Select the basis $\psi^{*}=\arg\max\left(L_{\psi }\right)$. This process adaptively tailors the wavelet analysis to each signal’s unique fault characteristics, as visualized in Figure 2.

Unlike traditional wavelet transforms that use a fixed basis function (e.g., Morlet), the adaptive mechanism in the AWLT dynamically selects the most informative basis based on energy and entropy metrics (Equation (7)). This ensures the time–frequency representation aligns with fault characteristics, enhancing the generator’s ability to synthesize realistic vibration patterns under variable operating conditions.

### 3.2. Adaptive Wavelet-like Transform Neural Networks

The signal can be transformed using wavelet transform to obtain a wavelet coefficient matrix, which inherently represents the values of the inner product between the wavelet, varying with scale and translation factors, and the signal. This reflects the frequency distribution of the signal at different times. Given the wavelet coefficient matrix and the wavelet function, the signal can also be reconstructed using the inverse wavelet transform. Inspired by autoencoders (AEs), the wavelet transform and inverse transform can be viewed as two mappings, which can be replaced by multi-layer neural networks through iterative learning, resulting in a wavelet-like transform neural network. Neural networks excel at handling complex nonlinear relationships and can learn more complex mappings than traditional wavelet transforms through training, thus better adapting to the nonlinear characteristics in practical applications. Integrating wavelet-like transform neural networks into the GAN model can enhance the performance of the entire system, rather than focusing solely on the effect of a single step in the wavelet transform.

### 3.3. Adaptive Wavelet-like Transform Neural Network

**Figure 3:**
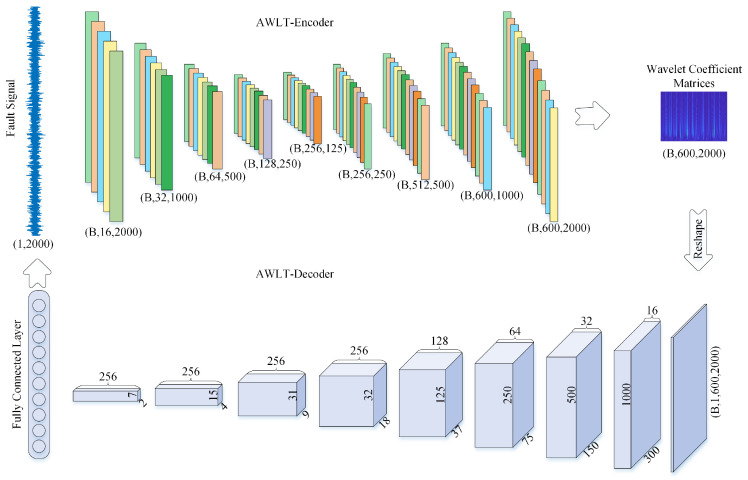
Architecture of the adaptive wavelet transform-like neural network.

A one-dimensional vibration signal can be transformed into a two-dimensional wavelet coefficient matrix through wavelet transform, which captures time–frequency characteristics of the signal. Conversely, the inverse wavelet transform reconstructs the original signal from this matrix. The proposed adaptive wavelet-like transform neural network (AWLT) implements this bidirectional mapping via an encoder–decoder architecture, as illustrated in Figure 3.

#### 3.3.1. AWLT-Encoder: Time-to-Time–Frequency Mapping

The encoder processes a fixed-length vibration signal x(n) of dimensions (B,1,2000), where *B* denotes the batch size, through a hierarchical 1D convolutional network:

- Input Layer: Raw signal (B,1,2000).
- Network Architecture: Hierarchical structure with downsampling and upsampling blocks. Detailed configurations are listed in Table 1. (Note: transposed convolutions include output padding adjustments to align dimensions).
- Output: A 2D wavelet coefficient matrix W(a,b) of dimensions (B,600,2000).

The encoder is optimized by minimizing the Mean Squared Error (MSE) between predicted and reference wavelet coefficients (Equation (8)).

#### 3.3.2. AWLT-Decoder: Time–Frequency-to-Time Mapping

The decoder reconstructs the original signal from the wavelet coefficient matrix W(a,b) through an inverse mapping:

- Input: Reshaped wavelet coefficients (B,1,600,2000).
- Feature Extraction Blocks: Eight 2D convolutional layers downsample the time–frequency representation. Detailed configurations are listed in Table 2. (Note: Output dimensions are given in (Channels, Length) format. All convolutional layers use ReLU activation and Batch Normalization).
- Reconstruction: The final feature map is flattened and passed through a fully connected layer to reconstruct the original signal $\hat{x}\left(n\right)$ with dimensions (B,1,2000).

The decoder is trained using MSE loss between $\hat{x}\left(n\right)$ and x(n), combined with L1–L2 regularization terms ($\lambda_{1}=\lambda_{2}=0.0001$) to enhance sparsity and prevent overfitting.

(8) $$\left\{\begin{array}{c} L_{en}=\frac{1}{MN}\sum_{i=1}^{M}\sum_{j=1}^{N}{\left(\hat{W}\left(a,b\right)-W(a,b)\right)}^{2}\\ L_{de}=\frac{1}{N}\sum_{j=1}^{N}{\left({\hat{x}}_{j}-x_{j}\right)}^{2}\\ T_{loss}=L_{en}+L_{de}+\lambda_{1}\sum_{k}\left|W_{k}\right|+\lambda_{2}\sum_{l}W_{l}^{2} \end{array}\right.$$

where $\hat{W}\left(a,b\right)$ is the wavelet coefficient matrix generated by the encoder. $\hat{x}$ is the signal reconstructed by the decoder. $\lambda_{1}$ and $\lambda_{2}$ are the L1 regularization weights. R1 and R2 are the weights in the neural network.

### 3.4. Architecture of AWLT-GAN

Typical GAN network structures usually include a generator and a discriminator. The differences between real data and generated data are computed by the discriminator, and the generator’s parameters are updated via backpropagation to learn the features of the real data. When the input to the discriminator is either real time-domain or frequency-domain data, the generator can only learn part of the real data’s features due to the limitations of the GAN model structure. By embedding an adaptive wavelet-like transform neural network into the generator of the GAN, the generator first generates a wavelet coefficient matrix, which is then converted into a vibration signal. Correspondingly, two discriminators are constructed so that in one iteration, the discriminator can evaluate both the local time–frequency feature wavelet coefficient matrix and the time-domain feature vibration signal. Through backpropagation, the generator’s parameters are simultaneously updated, enabling the generated signal to possess more complete tim- domain and frequency-domain attributes.

**Figure 4:**
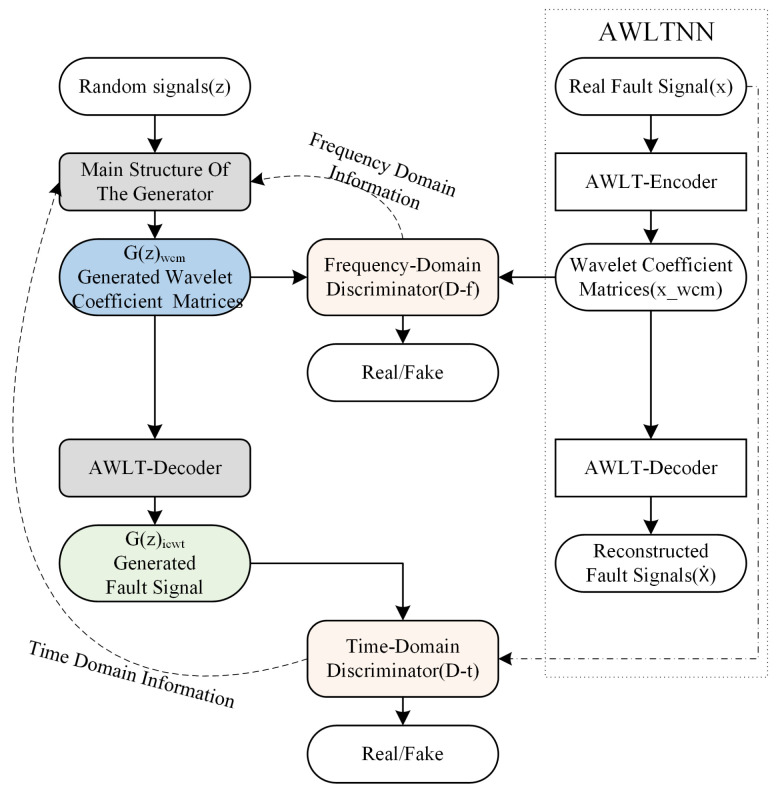
Schematic diagram of AWLT-GAN.

The structure of the AWLT-GAN is shown in Figure 4. It includes two discriminators and one generator. The generator consists of two parts: the main generator and the adaptive wavelet-like decoding layer. The two discriminators are the frequency-domain discriminator $D_{f}$ and the time-domain discriminator $D_{t}$. The frequency-domain discriminator’s role is to distinguish whether the wavelet coefficient matrix with frequency-domain characteristic information is transformed from a real signal or generated by the generator. The time-domain discriminator’s role is to distinguish whether the vibration signal with time-domain characteristic information is real or generated by the generator.

The generator of the AWLT-GAN is composed of two parts: the main structure of the generator is the same as the adaptive wavelet-like transform encoder, and the adaptive wavelet-like decoding layer structure is the same as the adaptive wavelet-like transform decoder. During training, the parameters of the wavelet-like decoding layer are fixed, and the parameters of the main structure are primarily updated. The loss function is as shown in Equation (9).

(9) $$L_{G}=E_{z_{wcm}∼p_{g-wcm}}\left[D_{f}\left(G\left(z_{wcm}\right)\right)\right]+E_{z∼p_{g}}\left[D_{t}\left(G\left(z\right)\right)\right]$$

where $p_{g-wcm}$ is the probability distribution of the wavelet coefficient matrix $G{\left(z\right)}_{wcm}$ obtained through noise *z*, $p_{g}$ is the probability distribution of the vibration signal G(z) generated by noise z, $D_{f}$ is the discriminator function in the frequency domain, and $D_{t}$ is the discriminator component in the time domain.

The constructed AWLT-GAN contains two discriminators with different functions: Time-Domain Discriminator ($D_{t}$): processes raw vibration signals to evaluate time-domain authenticity. Frequency-Domain Discriminator ($D_{f}$): analyzes wavelet coefficient matrices to assess time–frequency characteristics.

**Figure 5:**
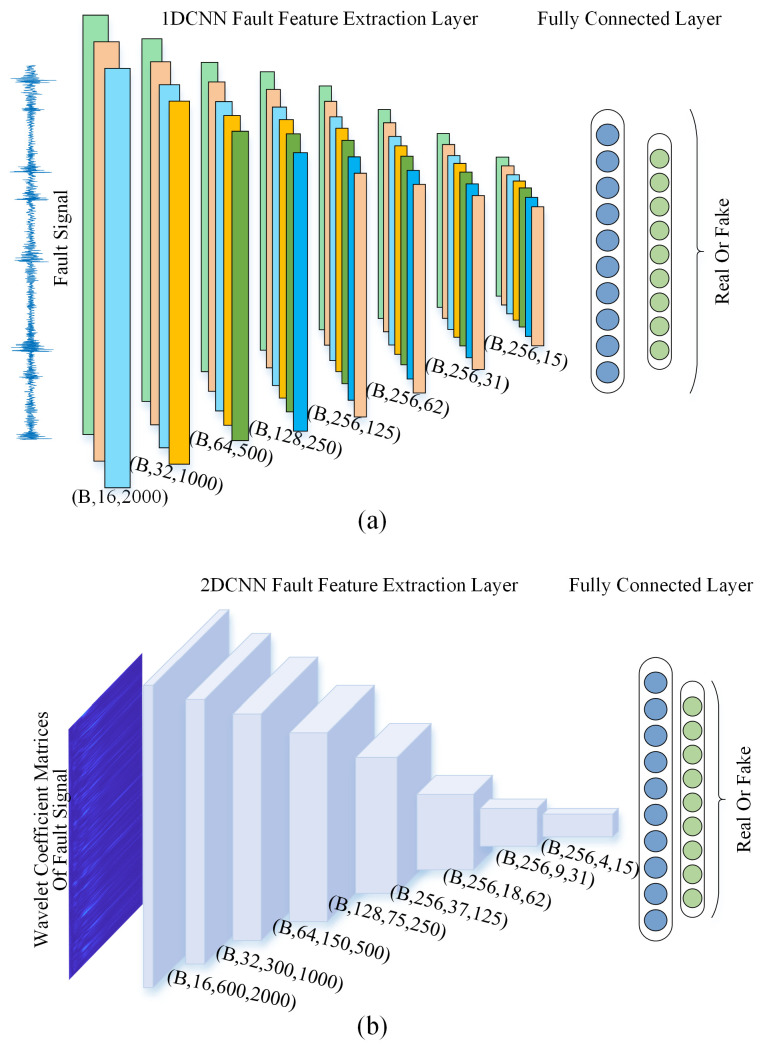
Schematic diagram of two discriminators. (**a**) Time-domain discriminator. (**b**) Frequency-domain discriminator.

To stabilize adversarial training, both discriminators employ the Wasserstein distance with a gradient penalty term (Equations (10) and (11)), effectively mitigating gradient vanishing and mode collapse. The network architectures are illustrated in Figure 5, with detailed layer configurations provided below.

(10) $$\left\{\begin{array}{c} L_{D_{t}}=-E_{x∼p}\left[D_{t}\left(x\right)\right]+E_{z∼p_{g}}\left[D_{t}\left(G\left(z\right)\right)\right]+\gamma_{GP_{t}}\\ \gamma_{GP_{t}}=\lambda E_{{\hat{x}}_{t}∼p_{{\hat{x}}_{t}}}\left[{\left({∥\nabla_{{\hat{x}}_{t}}D\left({\hat{x}}_{t}\right)∥}_{2}-1\right)}^{2}\right] \end{array}\right.$$

where *p* is the distribution of the sample *x*, $\gamma_{GP_{t}}$ is the gradient penalty term for the time-domain discriminator, and ${\hat{x}}_{t}$ denotes the uniform sampling of the time-domain generated samples and the real samples.

(11) $$\left\{\begin{array}{cc} L_{D_{f}}= & -E_{x_{wcn}∼p_{d-wcm}}\left[D_{f}\left(x_{wcm}\right)\right]\\ \; & +E_{z_{wcm}∼p_{g-wcm}}\left[D_{f}\left(G\left(z_{wcm}\right)\right)\right]+\gamma_{GP_{f}}\\ \gamma_{GP_{f}}= & \lambda E_{{\hat{x}}_{f}∼p_{{\hat{x}}_{f}}}[(∥\nabla_{{\hat{x}}_{f}}D\left({\hat{x}}_{f}\right){∥}_{2}-1{)}^{2}] \end{array}\right.$$

where $p_{d-wcm}$ is the distribution of the wavelet coefficient matrix $x_{wcm}$ obtained through wavelet transform of the sample *x*, $\gamma_{GP_{f}}$ is the gradient penalty term for the frequency-domain discriminator, λ is the gradient penalty coefficient, ${\hat{x}}_{f}$ denotes the uniform sampling of the frequency-domain generated samples and the real samples, $\nabla_{{\hat{x}}_{f}}D\left({\hat{x}}_{f}\right)$ is the gradient of the frequency-domain discriminator’s output results for ${\hat{x}}_{f}$, and ${∥\cdot ∥}_{2}$ is the L2 norm.

#### 3.4.1. Time-Domain Discriminator ($D_{t}$)

The discriminator $D_{t}$ takes a vibration signal x(t) of dimensions (B,1,2000) as input and processes it through a hierarchical 1D convolutional network:

- Key Components: All convolutional layers follow the sequence Conv1D → BatchNorm → LeakyReLU (α=0.2) → MaxPool1d; max pooling is carried out with window size = 2 and stride = 2. The final output uses Global Average Pooling + Linear layer.
- Network Architecture: The hierarchical structure with explicit padding control and progressive downsampling is shown in Table 3.
- Objective: Minimizes the Wasserstein distance between real and generated sample distributions, regulated by the gradient penalty term in Equation (10).

#### 3.4.2. Frequency-Domain Discriminator ($D_{f}$)

The discriminator $D_{f}$ operates on wavelet coefficient matrices W(a,b) of dimensions (B,1,600,2000), extracting joint time–frequency features:

- Key Components: All convolutional layers follow the sequence Conv2D → BatchNorm → LeakyReLU (α=0.2) → AdaptiveMaxPool2d; max pooling is carried out with output size = (H//2, W//2) after each convolution. The final output uses Global Average Pooling + Linear layer.
- Network Architecture: The hierarchical structure with progressive downsampling is shown in Table 4.
- Objective: Minimizes the Wasserstein distance between real and generated time–frequency distributions, regulated by the gradient penalty term in Equation (11).

The AWLT-GAN employs a dual-discriminator design to enforce fidelity in both time and frequency domains. The time-domain discriminator ($D_{t}$) evaluates the raw vibration signals, promoting accurate waveform reconstruction and temporal pattern learning. In contrast, the frequency-domain discriminator ($D_{f}$) analyzes the corresponding wavelet coefficient matrices (WCMs), guiding the generator to preserve time–frequency structures essential for fault characterization.

During training, the generator receives simultaneous feedback from both discriminators, and its parameters are updated accordingly in a joint optimization scheme. This strategy encourages the generation of signals that are not only realistic in the time domain but also maintain spectral integrity in the frequency domain. This dual-domain supervision enables AWLT-GAN to outperform conventional GANs with a single discriminator, especially in capturing complex fault signatures embedded in both domains.

### 3.5. Ensemble Learning Fault Diagnosis Model

Ensemble learning constructs several models with different characteristics and aggregates them according to a certain combination strategy, forming a model that has better generalization and accuracy than a single model. For time-domain vibration signals, one-dimensional convolution processes the signals by computing correlations in one dimension only, resulting in relatively low computation, faster processing, and more effective capture of contextual information along the time dimension, thus better extracting the time-domain features of the signal. For spectrograms obtained through wavelet transform, two-dimensional convolution can simultaneously capture both time and frequency information, comprehensively understanding the time–frequency characteristics of the signal and thus better extracting frequency-domain features.

To fully utilize both the time-domain and frequency-domain features of bearing fault signals and further enhance the effectiveness of the fault diagnosis model, this paper constructs two fault diagnosis models. The first model, based on one-dimensional convolution, primarily processes time-domain signals to extract time-domain features for fault diagnosis. Its feature extraction backbone is similar to the time domain discriminator of AWLT-GAN, utilizing one-dimensional convolutional modules, followed by two fully connected layers with a softmax activation function to output bearing fault types. To prevent overfitting, a dropout layer with a parameter set to 0.3 is added between the two fully connected layers. The second model, based on two-dimensional convolution, primarily processes the spectrograms obtained from the wavelet transform. Its feature extraction backbone is similar to the frequency-domain discriminator of AWLT-GAN, also using two-dimensional convolutional modules for feature extraction, with the remaining parts being similar to the first model. Finally, the two fault diagnosis models are combined through soft voting. In this study, an equal-weighted soft voting strategy was adopted, where the output probabilities of the time-domain and frequency-domain classifiers were averaged (i.e., weight = 0.5 for both). This simple and effective approach was chosen based on preliminary experiments, which showed no significant performance gain from weight tuning.

### 3.6. Workflow of Proposed Method

**Figure 6:**
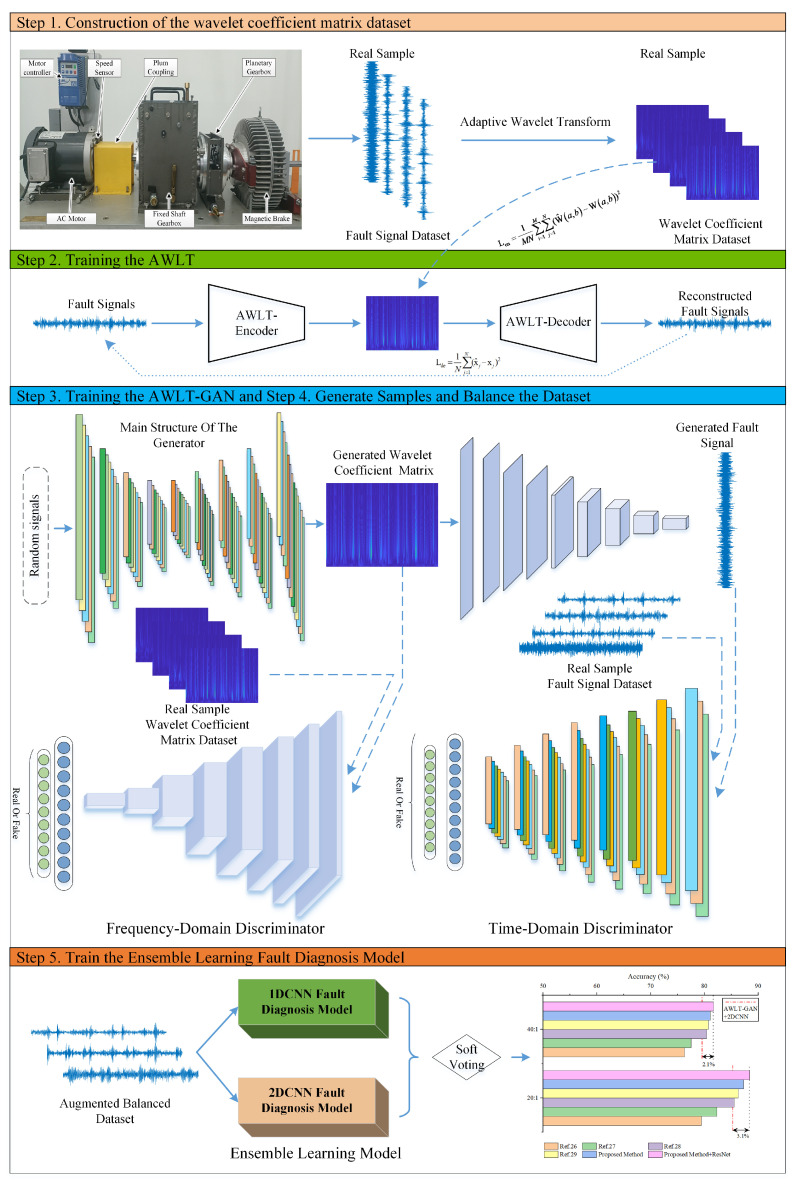
Flowchart of proposed bearing fault diagnosis method using AWLT-GAN and ensemble learning.

The workflow of the bearing fault diagnosis method based on AWLT-GAN and ensemble learning is shown in Figure 6, which includes the following main steps:

Step 1. Construction of the wavelet coefficient matrix dataset. Perform wavelet transform on the vibration signals using different wavelet basis functions, and select the wavelet coefficient matrix corresponding to the maximum $L_{\varphi }$ as the output result of the current vibration signal’s wavelet transform.

Step 2. Training the AWLT. Use real data as input to train the encoder and decoder of the adaptive wavelet-like transform neural network, determining the network parameters.

Step 3. Training the AWLT-GAN. During training, fix the parameters of the generator’s decoding layer and only update the parameters of the main generator network. To avoid overfitting of the generator and ensure the quality of generated data, iterate the discriminator and generator at a 2:1 ratio within one training cycle.

Step 4. Generate Samples and Balance the Dataset. Use the trained AWLT-GAN network to generate bearing fault data, expanding and balancing the dataset.

Step 5. Train the ensemble learning fault diagnosis model. Use the balanced dataset to separately train the time-domain and frequency-domain fault diagnosis models, and then determine the bearing fault types through a soft voting method.

## 4. Experiment I: Evaluation of AWLT-GAN

### 4.1. Test Rig and Experimental Data

**Figure 7:**
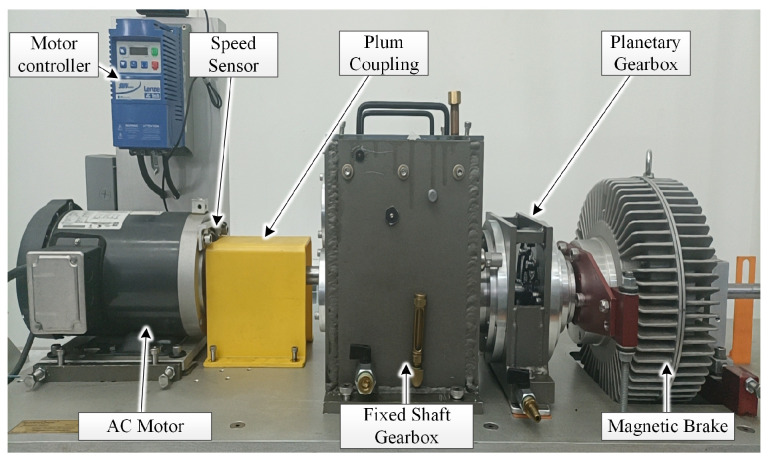
Comprehensive bearing fault diagnosis test rig.

To evaluate the performance of the AWLT-GAN-based bearing fault diagnosis model, we conducted tests using a comprehensive bearing fault diagnosis test rig. The structure of the test rig is shown in Figure 7. The motor controller controls the speed of the drive motor, which is connected to the planetary gearbox via a two-stage parallel shaft gearbox. The test bearing is mounted at the end of the second shaft of the parallel shaft gearbox. The bearing parameters are listed in Table 5. The acceleration sensor used in the experiments is the PCB 333B40 model, which has a measurement range of ±10 g and a resolution of 5 μg. The sensor is positioned in a radial vertical orientation on the bearing pedestal of the second shaft of the fixed shaft gearbox. There are three types of faulty bearings: inner ring fault, outer ring fault, and rolling element fault. During the tests, the motor speeds were set to 1000 rpm, 1500 rpm, and 2000 rpm, with a sampling frequency of 20,480 Hz.

Considering signal characteristics, model size, and hardware resources, the collected signals were normalized and segmented into datasets using a sliding window (see Table 6). The sliding window length was set to 2000, with a step size of 500.

### 4.2. Comparison Models

To demonstrate the superiority of the AWLT-GAN model, we constructed several comparison models based on methods referenced in the literature. The T-GAN model, primarily learning time-domain information, was constructed based on the method in [11]. During training, the model used real one-dimensional fault signals as inputs to the discriminator, which evaluated the authenticity of real and generated samples, calculated losses, and updated the generator accordingly. The FFT-GAN model, primarily learning frequency-domain information, was constructed based on the method in [16]. The model used spectrum information obtained from the Fourier transform directly as input to the discriminator, which evaluated the authenticity of real and generated samples, calculated losses, and updated the generator accordingly. Both the T-GAN and FFT-GAN feature extraction models were built using 1DCNNs, with network structures similar to wavelet encoders. The STFT-GAN model, primarily learning time–frequency-domain information, was constructed based on the method in [19]. The model used time–frequency spectrum information obtained from the short-time Fourier transform (STFT) directly as input to the discriminator, which evaluated the authenticity of real and generated samples, calculated losses, and updated the generator accordingly. The CWT-GAN model, primarily learning time–frequency-domain information, was constructed based on the method in [22]. The model used time–frequency spectrum information obtained from the Continuous Wavelet Transform (CWT) directly as input to the discriminator, which evaluated the authenticity of real and generated samples, calculated losses, and updated the generator accordingly. Both STFT-GAN and CWT-GAN models were built using 2DCNNs, with network structures similar to wavelet decoders. In the CWT-GAN model, the Morlet wavelet was chosen as the wavelet basis due to its capability to extract amplitude and phase information effectively in signals requiring high time–frequency localization.

### 4.3. Parameter Setting

To ensure the effective training of the AWLT-GAN, the learning rate for both the generator and discriminators is set to 0.0002, as a lower learning rate contributes to stabilizing the adversarial training process. The Adam optimizer is utilized for both networks, with parameters β1=0.5 and β2=0.999, ensuring smoother convergence and reducing the likelihood of mode collapse. The batch size is set to 32, and to guarantee sufficient training, the model is trained for 2000 epochs. During each epoch, the discriminators are updated twice for every update of the generator to prevent generator overfitting. A gradient penalty term is introduced in both discriminators to enforce Lipschitz continuity, which helps prevent mode collapse and vanishing gradients. The gradient penalty coefficient λ is set to 10. For the adaptive wavelet-like transform neural network (AWLTNN), the regularization terms are configured as $\lambda_{1}=\lambda 2=0.0001$ for L1 regularization. The learning rate for both the encoder and decoder is also set to 0.0002, with the Adam optimizer applied, maintaining the parameters β1=0.5 and β2=0.999. These parameter configurations are applied consistently across the generator, AWLT encoder–decoder, and both discriminators for stable adversarial training.

### 4.4. Quality Evaluation of AWLT-GAN-Generated Samples

#### 4.4.1. Quality Evaluation Based on Time-and-Frequency Spectrum

**Figure 8:**
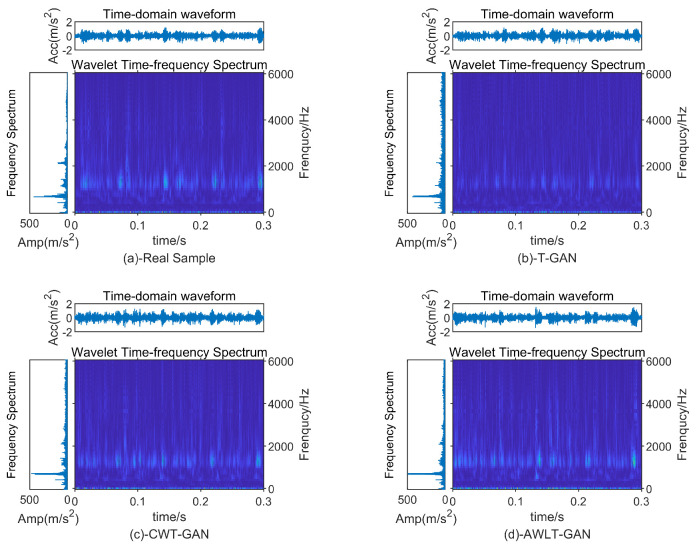
Bearing outer−ring signal time−and−frequency spectrum.

To visually evaluate the quality of the signals generated by AWLT-GAN, vibration signals from the outer ring of the bearing at 1500 rpm were selected for comparison. The comparative models used were T-GAN, which is based on time-domain information, and CWT-GAN, which is based on time–frequency information. After the models were trained, the generated signals were preprocessed, with the results shown in Figure 8. Overall, all three GAN models were able to generate signals similar to the real samples, though subtle differences were observed in the time–frequency-domain representations.

The signal generated by T-GAN exhibits impact characteristics similar to the real sample in the time-domain waveform, but it contains more noise. While its frequency characteristics in the spectrum reflect the signal features, they are less distinct compared to the real signal, and overall, the signal appears less clean than the real sample. The signal generated by CWT-GAN performs well in both the time-domain waveform and the spectrum. Impact characteristics similar to the real sample can also be observed in the time-domain waveform, although it contains slightly more noise compared to T-GAN. In the spectrum, the characteristic frequencies are more prominent than those in T-GAN, and the other frequency components appear cleaner. AWLT-GAN also performs well in both the time-domain waveform and the spectrum. Although there is some deviation from the real sample, the difference is not visually significant.

From the above observations, all three GAN models generate signals that are similar to the real samples, which can be attributed to the robust performance of the GAN models. However, the subtle differences also highlight the characteristics of each model. The T-GAN model excels in generating time-domain information, the CWT-GAN performs better in the frequency domain, while the AWLT-GAN model shows superior performance in both time-domain and frequency-domain generation.

#### 4.4.2. Quality Evaluation Based on Statistical Metrics

To accurately assess the quality of samples generated by AWLT-GAN, a quantitative method was used. We randomly selected one real sample and one generated sample with the same working condition and defect type, respectively, to form a sample group. This process was repeated 50 times consecutively, and the average quantitative parameter was calculated. For T-GAN and FFT-GAN, the learned samples are real one-dimensional time-domain and frequency-domain signals, and the output is also one-dimensional signals. Therefore, the Pearson Correlation Coefficient (PCC) (12) [38] and Cosine Similarity (CS) (13) [39] were used to evaluate the quality of the generated samples.

(12) $$PCC\left(x,y\right)=\frac{\frac{1}{n}\sum_{i=1}^{n}\left(x_{i}-\bar{x}\right)\left(y_{i}-\bar{y}\right)}{\sqrt{\frac{1}{n}\sum_{i=1}^{n}{\left(x_{i}-\bar{x}\right)}^{2}}\cdot \sqrt{\frac{1}{n}\sum_{i=1}^{n}{\left(y_{i}-\bar{y}\right)}^{2}}}$$

(13) $$S\left(x,y\right)=\frac{\sum_{i=1}^{n}x_{i}y_{i}}{\sqrt{\sum_{i=1}^{n}x_{i}^{2}}\times \sqrt{\sum_{i=1}^{n}y_{i}^{2}}}$$

where $x_{i}$ and $y_{i}$ are the values of samples *x* and *y* at index *i*, respectively, $\bar{x_{i}}$ and $\bar{y_{j}}$ are the means of samples *x* and *y*, respectively, and *n* is the length of the sample.

The higher the PCC and CS values, the more similar the generated samples are to the real samples.

For STFT-GAN and CWT-GAN, the learned samples are two-dimensional time–frequency matrices obtained from Fourier and wavelet transforms of one-dimensional time-domain signals, and the output is also two-dimensional time–frequency matrices. Hence, the Structural Similarity Index (SSIM) (14) [40] and Peak Signal-to-Noise Ratio (PSNR) (15) were used to evaluate the quality of the generated samples.

(14) $$SSIM\left(x,y\right)=\frac{\left(2\mu_{x}\mu_{y}+C_{1}\right)\left(2\sigma_{xy}+C_{2}\right)}{\left(\mu_{x}^{2}+\mu_{y}^{2}+C_{1}\right)\left(\sigma_{x}^{2}+\sigma_{y}^{2}+C_{2}\right)}$$

where $\mu_{x}$ and $\mu_{y}$ are the means of samples *x* and *y*, $\sigma_{xy}$ is the covariance between samples *x* and *y*, and $\sigma_{x}$ and $\sigma_{y}$ are the variances of samples *x* and *y*. $c_{1}$ is a constant, designed to avoid instability when ${\mu_{x}}^{2}+{\mu_{y}}^{2}$ is very close to zero. $c_{2}$ is a constant, designed to avoid instability when ${\sigma_{x}}^{2}+{\sigma_{y}}^{2}$ is very close to zero.

(15) $$PSNR\left(x,y\right)=10\log_{10}\frac{255\times 255}{\frac{1}{m\times n}\sum_{i=1}^{m}\sum_{i=1}^{n}{\left(x\left(i,j\right)-y\left(i,j\right)\right)}^{2}}$$

where *m* and *n* are the number of rows and columns of the sample matrix *x* and *y*, respectively.

Higher values of SSIM and PSNR indicate higher similarity between the generated samples and the real samples. Additionally, since AWLT-GAN generates one-dimensional vibration signals, SSIM and PSNR evaluations are performed after wavelet transform.

These metrics were selected not only for their relevance to signal similarity but also due to their widespread use in generative signal and image evaluation tasks, ensuring consistent and interpretable assessments across domains.

The results presented in Table 7 indicate that the generated samples exhibited a Pearson Correlation Coefficient (PCC) of 0.9287 and a Cross-Spectrum (CS) of 0.9921 in the time domain, reflecting a high degree of similarity with the real samples. Additionally, in the frequency domain, the Structural Similarity Index Measure (SSIM) and Peak Signal-to-Noise Ratio (PSNR) for the real and generated wavelet coefficient matrices were 0.8329 and 21.8462, respectively, which demonstrates the AWLT-GAN’s successful capture of the key features of the real data across both domains. This effectiveness is largely attributed to the model’s simultaneous learning of the time-domain and frequency-domain properties of the real data during the training process.

#### 4.4.3. Quality Evaluation Based on TRTS

The Train-Real Test-Synthetic (TRTS) method is a widely used approach to evaluate the authenticity of generated data [41]. In this evaluation, a fault diagnosis model is trained on real bearing fault data and then tested on synthetic data produced by GAN models. A high TRTS accuracy indicates that the generated data share a feature distribution close to the real data, thus enabling the trained model to maintain robust diagnostic performance on synthetic samples. In our implementation, a 1D-CNN-based model is used for time- and frequency-domain signal classification, while a 2D-CNN-based model is used for time–frequency representations. Since AWLT-GAN generates one-dimensional vibration signals, we also apply wavelet transforms when evaluating with 2D-CNN to ensure a fair comparison.

**Figure 9:**
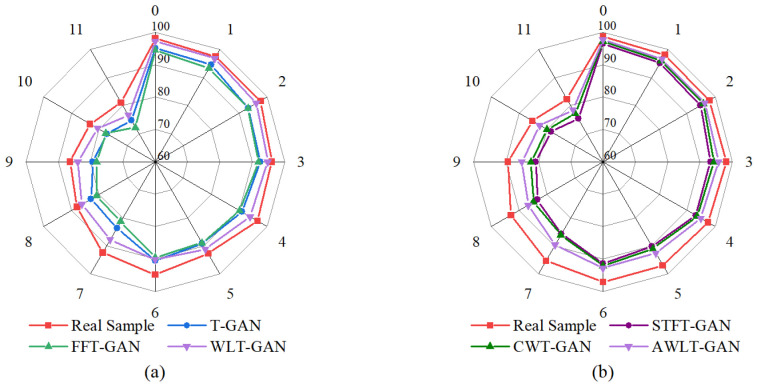
TRTS results with different diagnosis models. (**a**) Based on 1DCNN. (**b**) Based on 2DCNN.

The average diagnostic accuracy results are shown in Figure 9. It can be seen that, compared to T-GAN and FFT-GAN, which only utilize time-domain or frequency-domain information, AWLT-GAN-generated data perform better in 1DCNN-based classification. This is because AWLT-GAN considers both time-domain and frequency-domain attributes, making the generated samples more authentic. Compared to STFT-GAN and CWT-GAN, which use time–frequency-domain information, AWLT-GAN-generated data also perform better in 2DCNN-based classification. This is because real vibration data lose some features during STFT or CWT transformations, while AWLT-GAN compensates for this deficiency to some extent by simultaneously learning time-domain and frequency-domain features.

It is also observed that the fault location significantly impacts classification accuracy. The classification accuracy for normal state and outer ring faults is higher, while the classification accuracy for rolling element fault is the lowest. This variance in performance is attributed to the distinct signal signatures associated with each fault type. Rolling element faults often produce subtler and more complex vibration patterns that are challenging to discern, especially under noisy operational conditions. Observing from the perspective of rotational speed, the classification accuracy is higher at lower speeds (1000 rpm) and lower at higher speeds (2000 rpm). This is because as the speed increases, the noise generated by surrounding components increases, making the fault characteristics less noticeable in the real signal, which affects the quality of the generated signal and subsequently the performance of the fault diagnosis model. Additionally, the minimum fault identification accuracy of the fault diagnosis model in the experiments is 72.2%, which also indirectly verifies the feasibility of using GANs to handle imbalanced bearing fault data.

#### 4.4.4. Quality Evaluation Based on TSTR

The Train-Synthetic Test-Real (TSTR) method is commonly used to assess the diversity of generated data [41]. In this approach, a diagnostic model is trained solely on synthetic data and evaluated on real bearing fault data. A high TSTR accuracy suggests that the generated samples sufficiently cover the variability and feature distribution of real data, enabling the model to generalize well across real-world conditions. This metric reflects the extent to which the synthetic data encapsulate diverse fault characteristics, making it a key indicator of data diversity.

**Figure 10:**
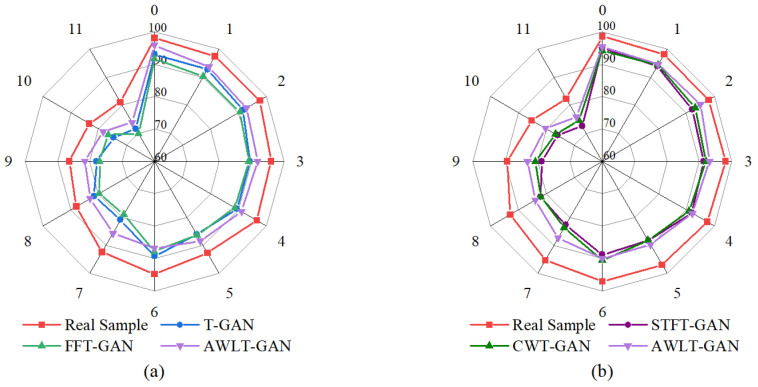
TSTR results with different diagnosis models. (**a**) Based on 1DCNN. (**b**) Based on 2DCNN.

The average diagnostic accuracy results are shown in Figure 10. Similar to the TRTS results, AWLT-GAN demonstrated superior signal diversity compared to other methods due to its simultaneous learning of the time-domain and frequency-domain characteristics of real data. This advantage is due to the fact that, although other GAN models can learn the time-domain or frequency-domain characteristics of real data, the limitations of the GAN model structure and the signal preprocessing methods result in a feature distribution after extraction that is still less than that of the real data, which is not conducive to constructing an effective fault diagnosis model.

#### 4.4.5. Quality Evaluation Based on Fault Diagnosis Under Different Conditions

**Figure 11:**
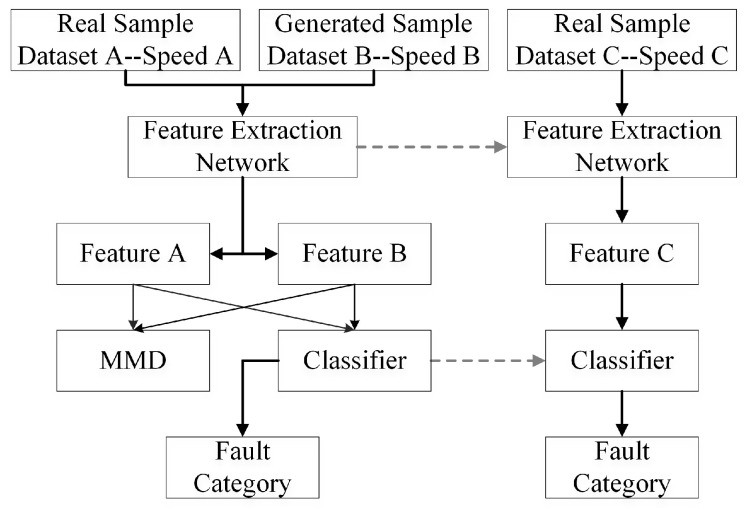
Method for evaluating generated sample quality based on fault diagnosis under different conditions.

As observed from the results in TRST and TSTR, the fault identification accuracy for the same defect generally decreases with increasing rotational speed. This observation may be related to the increased complexity of the signal under high-speed rotation conditions, which is caused by fluctuations in signal modulation and noise levels, affecting the extraction and recognition of fault features. However, based on the bearing fault mechanism model, the characteristic frequency of a bearing fault mainly varies with the rotational speed, meaning that the same fault has similar high-dimensional feature distributions at different rotational speeds [42,43]. Utilizing this characteristic, we can adopt a different condition classification method to observe the differences in high-dimensional feature distributions of data generated by different GAN models and verify the ability of the GAN models to generate high-quality data. Similar to the previous validation method design, one-dimensional data use a 1DCNN model, two-dimensional data use a 2DCNN model, and AWLT-GAN-generated data still undergo wavelet transformation. The details about the different conditions are given in Table 8, and the workflow of the fault diagnosis under different conditions is shown in Figure 11, which includes the following main steps:

Step 1. Use real dataset B at rotational speed B to train the GAN network and generate bearing dataset B.

Step 2. Use real dataset A at rotational speed A and the generated bearing dataset B to train the feature extraction network and classifier.

Step 3. Validate the constructed fault diagnosis model with real dataset C at rotational speed C.

**Table 8:** Details about the different conditions.

Task Label	Real Sample A Speed/rpm	Generated Sample B Speed/rpm	Real Sample C Speed/rpm
Task1	1000	1500	2000
Task2	1500	1000	2000
Task3	1000	2000	1500
Task4	2000	1000	1500
Task5	1500	2000	1000
Task6	2000	1500	1000

**Figure 12:**
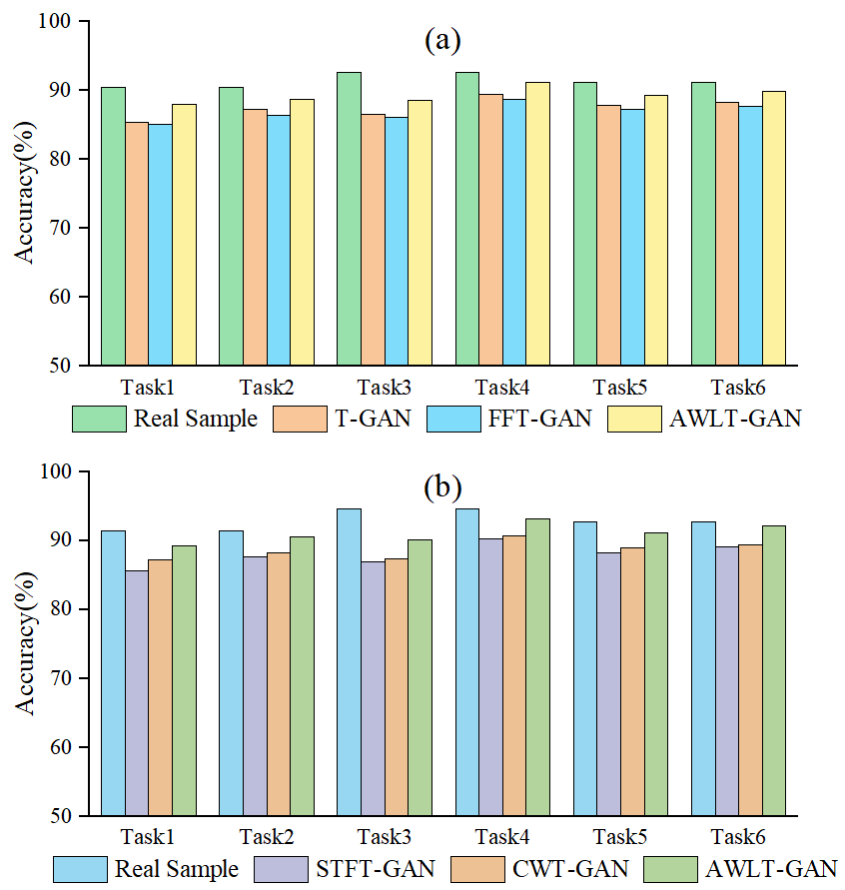
Result of fault diagnosis under different conditions. (**a**) Based on 1DCNN. (**b**) Based on 2DCNN.

The results of the different condition classification experiment are shown in Figure 12. The results indicate that when real data are used instead of generated dataset B in the multi-classification experiment, the average accuracy exceeds 90%, demonstrating that datasets under different conditions have similar feature distributions in high-dimensional feature space. Therefore, this method can be used to verify the quality of generated data and assess the gap between them and real data.

From the Figure 12, it can be seen that using generated data to train the model affects the classification accuracy to some extent. This is mainly because the generated data still lag behind real data in terms of authenticity and diversity. Nevertheless, the minimum accuracy for fault classification still reaches 85%, reflecting the ability of the GAN-based bearing fault signal generation data model to learn and capture the characteristics of real signals. Additionally, the result also shows that the STFT-GAN and CWT-GAN models, based on time–frequency features, perform better overall than the T-GAN and FFT-GAN models, which are based on single time-domain or frequency-domain features. This is partly due to the stronger feature extraction capability of 2DCNN and partly because the time–frequency-based GAN models generate data containing more real characteristics.

Overall, the AWLT-GAN model demonstrated high classification accuracy among all GAN models, with its fault diagnosis results closely matching those obtained using real data. This indicates that the data generated by AWLT-GAN have high consistency with real data in high-dimensional feature space, highlighting the advantage of AWLT-GAN in learning more comprehensive features from both the time and frequency domains.

### 4.5. AWLT Performance Evaluation

**Figure 13:**
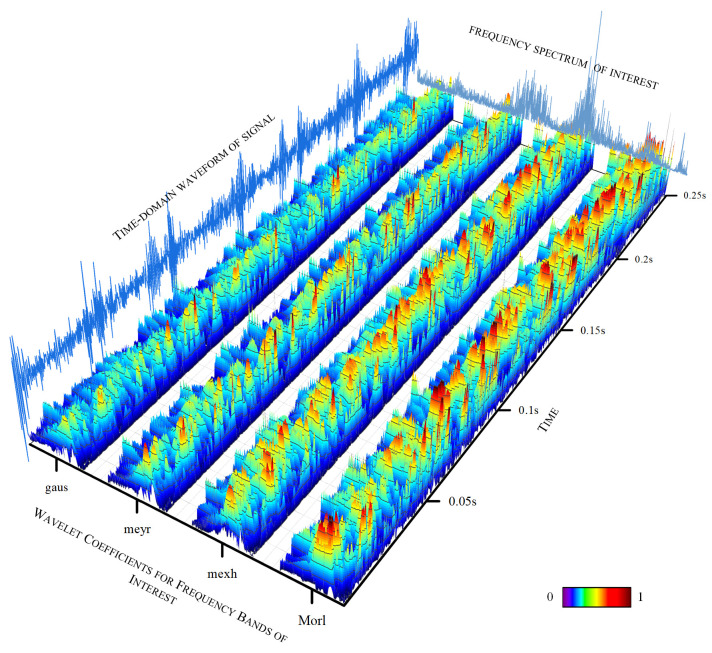
Wavelet time–frequency spectrum for outer ring faults using different wavelet basis functions.

**Figure 14:**
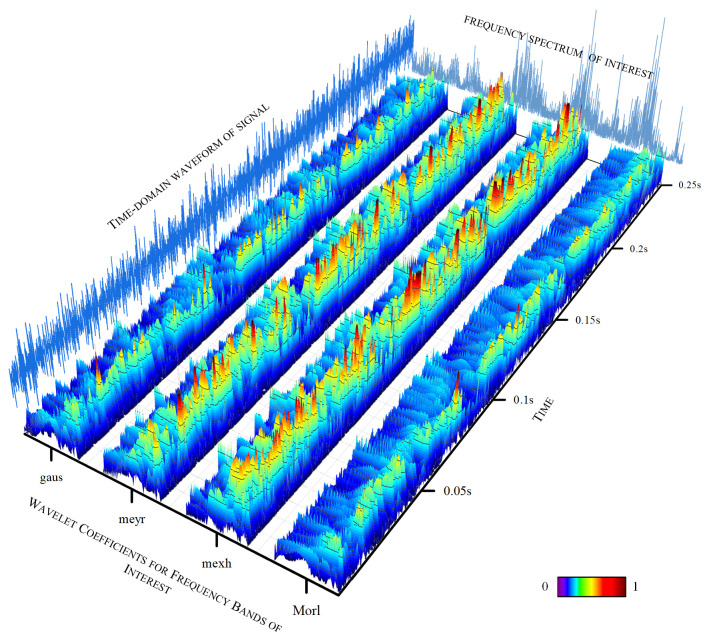
Wavelet time–frequency spectrum for ball faults using different wavelet basis functions.

The experiments above demonstrate that AWLT-GAN has significant advantages over conventional GANs. On one hand, the GAN model was optimized; on the other hand, a method capable of adaptively selecting wavelet basis functions was used. This study selected signals of outer ring and rolling element faults of bearings under 1500 rpm conditions. Four different wavelet basis functions were used to perform wavelet transforms on the signals, and the obtained wavelet coefficients were normalized, as shown in Figure 13 and Figure 14. From the figures, it can be seen that different wavelet basis functions have different time–frequency characteristics for the same signal. Their performance varies in both the time domain and the frequency domain, thus affecting the signal analysis results.

Figure 13 shows the results of analyzing the outer ring fault signal of the bearing using different signal processing methods. From the time-domain waveform, it can be seen that the signal has pronounced impulsive characteristics on the time scale. The Morlet wavelet, due to its excellent time-domain localization characteristics, responds well to oscillating signals and accurately captures the transient changes in the outer ring fault signal. From the three-dimensional trend of wavelet coefficient variations, it can be observed that the wavelet coefficients at the peaks on the time axis are prominent, effectively reflecting the impulsive characteristics of the signal.

Figure 14 shows the results of analyzing the rolling element fault signal of the bearing using different signal processing methods. From the time-domain waveform, it can be seen that the overall signal-to-noise ratio is relatively low. The Mexh wavelet, with its shape similar to the second derivative, is sensitive to abrupt points in the signal (such as impacts and vibration initiation points), and can effectively extract fault characteristic information from signals with a low signal-to-noise ratio. Figure 13 and Figure 14 also further demonstrate that processing signals with different wavelet basis functions results in different fault characteristic information.

**Figure 15:**
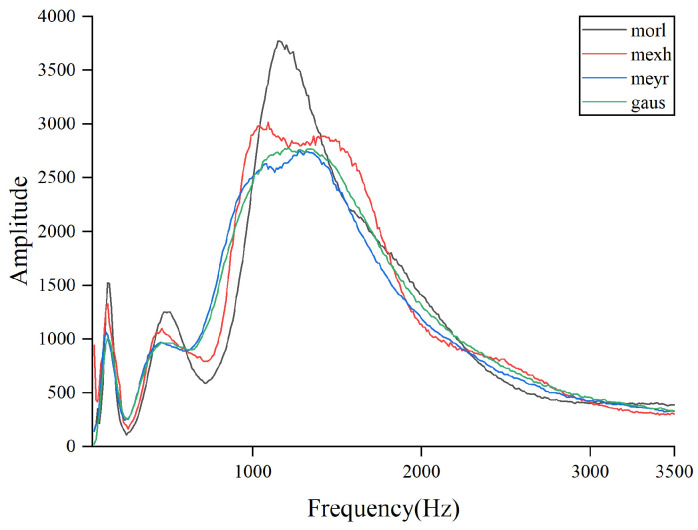
Product of wavelet coefficient energy and MRDE for outer ring faults at different center frequencies.

**Figure 16:**
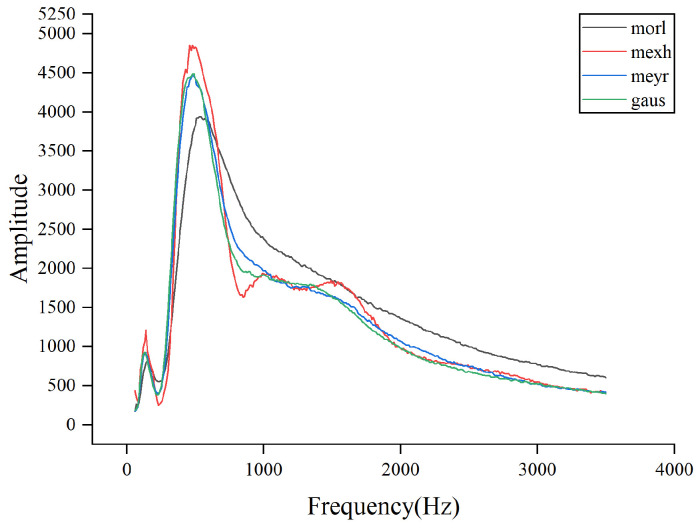
Product of wavelet coefficient energy and MRDE for ball faults at different center frequencies.

By calculating the product of wavelet coefficient energy and MRDE at different scale factors, the amount of fault characteristic information obtained after wavelet transformation can be better reflected. In Figure 15 and Figure 16, the combination of wavelet coefficient energy and MRDE can easily distinguish the ability of different wavelet basis functions to extract fault characteristics within the signal at the same center frequency. Observations show that the quantitative calculation results are consistent with the qualitative results of the three-dimensional diagrams, proving the feasibility of the proposed evaluation method.

To further verify the superiority of the AWLT method, and to confirm that using appropriate wavelet basis functions can obtain more prominent time–frequency characteristics of the signal, different wavelet basis functions were used to perform wavelet transformations on the signals, and the fault diagnosis model was trained with the optimal results shown in Table 9. The fault diagnosis model is also constructed based on a 2D CNN, and the model architecture is the same as in the previous experiments. The results indicate that, using the same fault signal dataset and network structure, signals processed based on the AWLT method allow the fault diagnosis model to acquire more information for fault type identification.

## 5. Experiment II: Imbalanced Fault Diagnosis

The aforementioned experiments indicate that AWLT-GAN can effectively improve the quality of generated data. However, it is important to note that these experiments were conducted with a certain amount of real data. In industrial practice, due to limitations in the installation environment of data collection hardware and the occasional and random nature of faults, only a limited amount of effective fault data can usually be collected. This results in a significant scale difference between normal and fault data, leading to imbalanced training dataset for fault diagnosis models and affecting the model’s accuracy. Common methods to address data imbalance include resampling and data augmentation. Among these, the GAN is widely used as an effective data augmentation method for handling the imbalance of bearing data.

**Figure 17:**
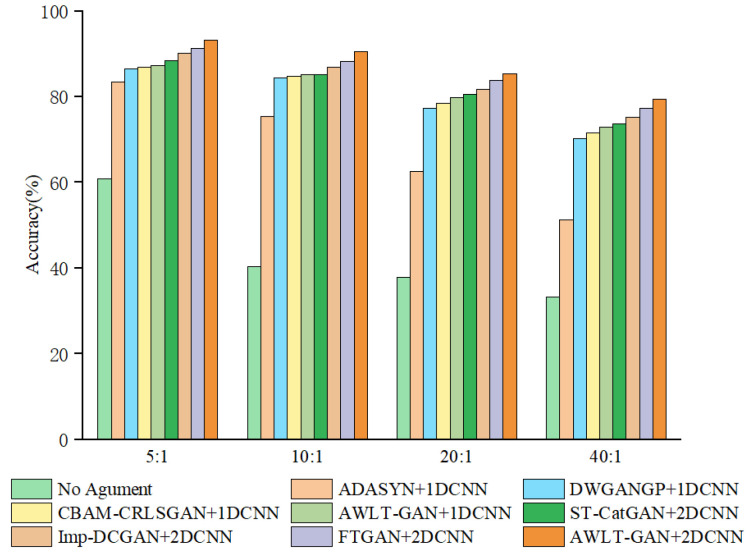
Result of imbalanced fault diagnosis.

To evaluate the performance of AWLT-GAN in dealing with imbalanced bearing data, a comparative experiment was set up. The experimental data settings are shown in Table 10. The compared models include the following: the ADASYN model [44] and DWGANGP model [15], focused on learning time-domain information, the CBAM-CRLSGAN model [16], focused on learning frequency-domain information, and the ST-CatGAN, Imp-DCGAN and FTGAN models [18,22,27], focusing on both time–frequency-domain information (the structure of the CBAM module in the CBAM-CRLSGAN data generation model is referenced from paper [45]). ST-CatGAN uses STFT for data preprocessing, while Imp-DCGAN uses CWT for data preprocessing. The FTGAN model also employs a dual discriminator structure, but it utilizes FFT for obtaining frequency-domain information. The construction of data generation models follows the methods in the relevant references, and the fault diagnosis models after data generation adopt the same 1DCNN or 2DCNN structure to evaluate the performance of different GAN generation methods in dealing with data imbalance. The experimental results are shown in Figure 17.

From the experimental results, it can be seen that as the data imbalance ratio increases, the performance of all diagnostic models decreases to varying degrees. Using data augmentation techniques can make the generated data have high-dimensional features similar to real data and increase a certain degree of diversity, thereby improving the accuracy of diagnostic models. However, due to the limitations of the generation model structure, these models have different performances in extracting real data features. As the data imbalance ratio increases, the number of real samples available for the generation model to learn features decreases, leading to a reduction in the effective features learned by some models, thus reducing the quality of the generated data.

The classic resampling method ADASYN, due to its generated samples not being diverse enough and being sensitive to noise, achieved only a 51.3% fault recognition rate at a 40:1 imbalance ratio. GAN-based models can learn signal features from real data, utilizing adversarial methods and deep neural networks to give GANs stronger feature learning capabilities. Even at a 40:1 imbalance ratio, with only 10 real samples, the diagnostic accuracy still reached 70.5%, demonstrating the potential of GAN methods in handling imbalanced bearing data and constructing effective fault diagnosis models.

Observing the results, it can be found that compared to DWGANGP and CBAM-CRLSGAN, which learn only time-domain or frequency-domain features, the ST-CatGAN, Imp-DCGAN, and FTGAN models, which learn time–frequency attributes, exhibit higher fault recognition rates in the test set after balancing the bearing dataset. This result is partly due to the superior feature extraction capabilities of 2DCNN and partly because the optimized GAN models can learn more feature information contained in real samples. However, at a 40:1 imbalance ratio, the performance of fault diagnosis models significantly decreases compared to the 20:1 ratio. This decrease is mainly due to the reduced number of real sample data available for the GAN model to learn, limiting the GAN model’s generation capabilities, thus reducing the quality of generated samples and ultimately affecting the accuracy of the fault diagnosis models. The FTGAN model also performs well; however, due to using the FFT method, its ability to obtain frequency-domain information is weaker than that of the CWT method when processing complex signals. AWLT-GAN demonstrates excellent performance when applied to fault diagnosis models utilizing both one-dimensional and two-dimensional convolutional approaches. This is due to its dual-discriminator architecture, which can learn high-dimensional feature information from both the time and frequency domains in one training session, making the generated signals closer to real signals in terms of authenticity and diversity. At a 40:1 imbalance ratio, when the amount of real data is 10, the AWLT-GAN model still has an accuracy of 79.6%, a 5.7% decrease compared to the 20:1 ratio.

Table 11 presents the precision, recall, and F1-Score of different methods under an imbalance ratio of 20:1. The results demonstrate that the proposed method achieves the highest values across all three metrics, verifying its superiority in diagnostic performance. Additionally, the experimental results demonstrate that AWLT-GAN effectively captures essential features from small sample data and generates high-quality bearing fault data. These generated data significantly contribute to improving the accuracy and stability of the fault diagnosis models based on AWLT-GAN.

## 6. Experiment III: Imbalanced Fault Diagnosis Using Ensemble Learning

According to the experimental results in fault diagnosis with imbalanced data, for the same set of generated data, the recognition accuracy is lower when using one-dimensional convolution for feature extraction directly on the signal compared to using two-dimensional convolution after preprocessing the signal. This phenomenon is due to two reasons: firstly, the time–frequency domain after transformation contains more fault feature information, and secondly, there is a difference in the feature extraction capabilities between one-dimensional and two-dimensional convolution. However, while wavelet transform can provide time–frequency information, it does not fully reflect the time–frequency characteristics of real samples. Additionally, the smoothing process in wavelet transform may introduce pseudo-features, causing subtle features in the time domain of the original signal to become less apparent after transformation. According to the results of imbalanced fault diagnosis, at a 40:1 imbalance ratio, although the fault recognition accuracy reached 79.6%, there were still almost 20 fault recognition errors, indicating that there is still significant room for improvement in fault recognition accuracy.

At the same time, one-dimensional convolution kernels perform convolution operations in only one direction. When processing time-series data collected by vibration sensors, they can effectively extract the intrinsic features of the signal from the time-series data, making them suitable for feature extraction of one-dimensional bearing time-series signals. Two-dimensional convolution, on the other hand, extracts features in two spatial dimensions simultaneously when processing two-dimensional data, better capturing the spatial information in two-dimensional spectral data, making it suitable for feature extraction of two-dimensional spectrograms. However, the data generated by AWLT-GAN contain both time-domain and frequency-domain information of real data. Using these data alone cannot fully exploit the advantages of AWLT-GAN. To more effectively utilize the information of bearing data, this study constructed an ensemble learning fault diagnosis model aimed at further improving fault diagnosis accuracy.

**Figure 18:**
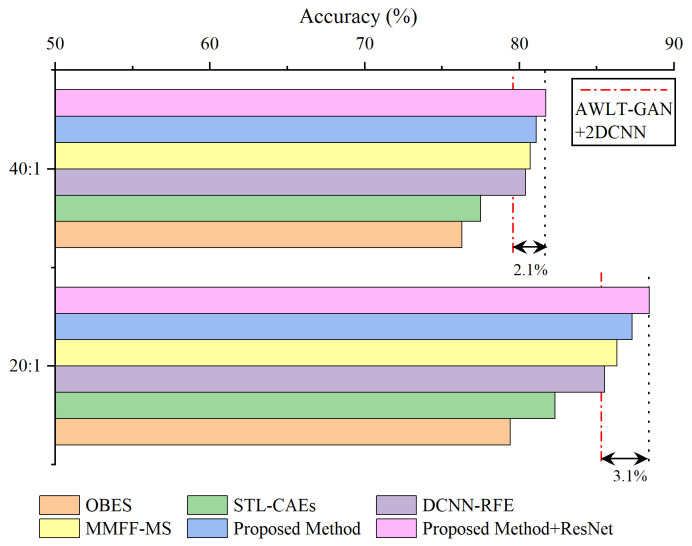
Ensemble learning-based fault diagnosis results for imbalanced data.

Based on the experiments in fault diagnosis with imbalanced data, datasets with imbalance ratios of 20:1 and 40:1 were selected, and the data generation models were used to balance the datasets. The comparison methods include OBES [29] and STL-CAEs [30], which utilize frequency-domain information, as well as DCNN-RFE [31] and MMFF-MS [32], which use time-frequency information. The ensemble learning method proposed in this paper is shown in Figure 6: one-dimensional time-domain signals are directly input into the 1DCNN fault diagnosis model, two-dimensional frequency-domain data, obtained from wavelet transform to obtain spectrograms, are input into the 2DCNN fault diagnosis model, and soft voting is used to integrate the classification probabilities to determine the final classification. The results are shown in Figure 18.

As shown in the results, the method MMFF-MS outperformed AWLT-GAN+2DCNN and other reference methods at imbalance ratios of 20:1 and 40:1. This superiority stems from two aspects: First, it extracts features from both the time domain and time-frequency domain, fully utilizing the features of the generated data. Second, it uses ResNet for feature extraction, which offers stronger capabilities than the network used in this paper. However, there are differences between MMFF-MS and the method proposed here. While MMFF-MS excels in feature extraction due to its use of ResNet for time-frequency information, the one-dimensional convolution-based method in this paper captures time-domain features more effectively. Additionally, MMFF-MS only extracts 28 features for classification, potentially underutilizing time-domain information. After optimizing our method by incorporating ResNet instead of 2DCNN, the ensemble learning approach in this paper achieved higher accuracy in fault classification compared to AWLT-GAN+2DCNN without ensemble learning. Specifically, at imbalance ratios of 20:1 and 40:1, the accuracy improved by 3.1% and 2.1%, respectively.

Compared to the method MMFF-MS, the method DCNN-RFE using ensemble learning focuses on repeatedly utilizing features from specific network layers, and its results also outperformed AWLT-GAN+2DCNN. However, due to the ineffective utilization of fault time-domain information, its fault diagnosis results were not as good as MMFF-MS and the method proposed in this paper. The methods OBES and STL-CAEs only used frequency-domain information, and although aided by ensemble learning methods, the improvement in fault diagnosis was not significant.

**Figure 19:**
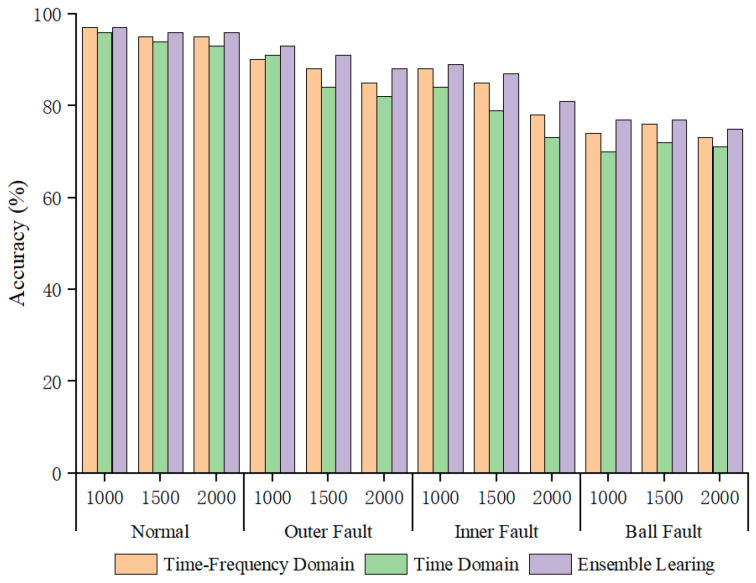
Result of various fault diagnosis methods at imbalance ratios of 20:1.

Overall, the ensemble learning method combining time-domain and frequency-domain classification results achieved higher fault recognition rates than models using time-domain or frequency-domain features alone, demonstrating the effectiveness of the ensemble learning method. To delve into the reasons behind the improved accuracy by integrated learning methods, we employed the AWLT-GAN approach to balance the dataset when the imbalance ratio reached 20:1. On this balanced dataset, we conducted three comparative experiments: the first using only time–frequency-domain information for fault diagnosis; the second using time-domain information alone for fault diagnosis; and the third applying an integrated learning method that utilizes both time–frequency information for fault diagnosis. The results are presented in Figure 19.

Observing the accuracy for different conditions and fault types, it can be seen that when fault signal features are obvious, such as normal bearings and outer ring faults, both 1DCNN and 2DCNN achieve good classification results. However, when the bearing fault signals are significantly influenced by their characteristics and surrounding components, such as rolling element faults, the quality of generated signals also decreases, the fault features extracted by deep neural networks become less distinguishable, and the classification accuracy when using them alone is not high. Using the ensemble learning method can improve the fault recognition accuracy of fault diagnosis models, as the soft voting strategy can integrate the feature information from both the time domain and frequency domain in high-dimensional space, thereby enhancing fault recognition accuracy.

## 7. Conclusions

To effectively address the imbalance in bearing fault data and improve the accuracy of fault diagnosis models, this paper proposes the AWLT-GAN method to generate fault data by fully utilizing both the time-domain and frequency-domain information of bearing fault signals, thus solving the data imbalance problem. On this basis, ensemble learning methods are adopted to further enhance the accuracy of fault diagnosis.

Compared to other methods, the proposed approach offers the following advantages: (1) it fully utilizes the time-domain and frequency-domain information of bearing fault signals, significantly improving diagnostic accuracy; (2) the generated samples are closer to real signals in terms of authenticity and diversity, providing a practical solution to the imbalance in bearing data.

Overall, the experiments and results in this paper confirm that the proposed method can effectively generate high-quality bearing fault signals and address the issue of data imbalance. However, the ensemble learning experiments suggest that more efficient feature extraction strategies could further enhance the performance of fault diagnosis models, which will be an important direction for future research. In addition, due to the use of dual discriminators, the stability of the AWLT-GAN model requires further improvement. Moreover, the limited recognition performance on rolling element faults warrants deeper investigation.

Furthermore, although the validation experiments include multiple rotating speeds, all data were collected under a fixed external load, representing relatively stable operating conditions. The capability of the proposed method to enhance fault data under non-stationary scenarios—such as fluctuating loads and variable speeds—was not comprehensively explored in this study. Given that non-stationary conditions can significantly alter vibration signal characteristics and pose major challenges in real-world fault diagnosis tasks, future work will explore extending the application of the AWLT-GAN framework to more diverse operating conditions, including varying load levels and speed changes. This extension will enable a more thorough evaluation of the model’s robustness and generalization capability in realistic industrial environments.

## Figures and Tables

**Table 1:** Layer configurations of the AWLT-Encoder.

Layer	Type	Input	Channels	Kernel	Stride	Output
1	Conv1D	(1, 2000)	1→16	5	1	(16, 2000)
2	Conv1D	(16, 2000)	16→32	5	2	(32, 1000)
3	Conv1D	(32, 1000)	32→64	5	2	(64, 500)
4	Conv1D	(64, 500)	64→128	5	2	(128, 250)
5	Conv1D	(128, 250)	128→256	5	2	(256, 125)
6	Transposed Conv1D	(256, 125)	256→256	5	2	(256, 250)
7	Transposed Conv1D	(256, 250)	256→512	5	2	(512, 500)
8	Transposed Conv1D	(512, 500)	512→600	5	2	(600, 1000)
9	Transposed Conv1D	(600, 1000)	600→600	5	2	(600, 2000)

**Table 2:** Configurations of the AWLT-Decoder.

Layer	Input	Channels	Kernel	Stride	Padding	Output
1	(1, 600, 2000)	1→16	3 × 3	2	1	(16, 300, 1000)
2	(16, 300, 1000)	16→32	3 × 3	2	1	(32, 150, 500)
3	(32, 150, 500)	32→64	3 × 3	2	1	(64, 75, 250)
4	(64, 75, 250)	64→128	3 × 3	2	(0, 1)	(128, 37, 125)
5	(128, 37, 125)	128→256	3 × 3	(2, 4)	(0, 1)	(256, 18, 32)
6	(256, 18, 32)	256→256	3 × 4	(2, 1)	(1, 1)	(256, 9, 31)
7	(256, 9, 31)	256→256	3 × 3	(2, 2)	(0, 0)	(256, 4, 15)
8	(256, 4, 15)	256→256	4 × 3	(2, 2)	(1, 0)	(256, 2, 7)

**Table 3:** Layer configurations of the time-domain discriminator ($D_{t}$).

Layer	Type	Input	Channels	Kernel/Stride	Padding	Output
1	Conv1D	(1, 2000)	1→16	5/1	2	(16, 2000)
2	Conv1D	(16, 1000)	16→32	5/1	2	(32, 1000)
3	Conv1D	(32, 500)	32→64	5/1	2	(64, 500)
4	Conv1D	(64, 250)	64→128	5/1	2	(128, 250)
5	Conv1D	(128, 125)	128→256	5/1	2	(256, 125)
6	Conv1D	(256, 62)	256→256	5/1	2	(256, 62)
7	Conv1D	(256, 31)	256→256	5/1	2	(256, 31)
8	Conv1D	(256, 15)	256→256	5/1	2	(256, 15)

**Table 4:** Layer configurations of the frequency domain-discriminator ($D_{f}$).

Layer	Type	Input	Channels	Kernel/Stride	Padding	Output
1	Conv2D	(1, 600, 2000)	1→16	3 × 3/1	1	(16, 600, 2000)
2	Conv2D	(16, 600, 2000)	16→32	3 × 3/1	1	(32, 600, 2000)
3	Conv2D	(32, 300, 1000)	32→64	3 × 3/1	1	(64, 300, 1000)
4	Conv2D	(64, 150, 500)	64→128	3 × 3/1	1	(128, 150, 500)
5	Conv2D	(128, 75, 250)	128→256	3 × 3/1	1	(256, 75, 250)
6	Conv2D	(256, 37, 125)	256→256	3 × 3/1	1	(256, 37, 125)
7	Conv2D	(256, 18, 62)	256→256	3 × 3/1	1	(256, 18, 62)
8	Conv2D	(256, 9, 31)	256→256	3 × 3/1	1	(256, 4, 15)

**Table 5:** Parameters of Bearing ER-16K.

Name	Value
radius of inner race/mm	25.4
pitch diameter/mm	38.51
number of balls	9
roller diameter/mm	7.94
contact angle (/°)	0

**Table 6:** Experimental dataset.

Label	Fault Location	Speed (rpm)	Sample Length	Sample Number
0	/	1000	2000	500
1	/	1500	2000	500
2	/	2000	2000	500
3	Outer Race	1000	2000	500
4	Outer Race	1500	2000	500
5	Outer Race	2000	2000	500
6	Inner Race	1000	2000	500
7	Inner Race	1500	2000	500
8	Inner Race	2000	2000	500
9	Ball	1000	2000	500
10	Ball	1500	2000	500

**Table 7:** Statistical metrics for quality assessment of various generative models.

Model	PCC	CS	SSIM	PSNR
T-GAN	0.9193	0.9873	/	/
FFT-GAN	0.8945	0.9782	/	/
STFT-GAN	/	/	0.8137	16.4432
CWT-GAN	/	/	0.8213	17,6921
AWLT-GAN	0.9287	0.9921	0.8329	21.8462

**Table 9:** Fault diagnosis results using different wavelet basis functions.

Wavelet Bias Function	Accuracy (%)
morl	92.4
mexh	89.7
meyr	91.4
gaus	87.6
AWLT	94.1

**Table 10:** Details of the dataset for imbalanced fault diagnosis at imbalance ratios of 20:1.

Imbalance Ratio	5:1	10:1	20:1	40:1
Real Normal Sample	400	400	400	400
Real Fault Sample Per Class	80	40	20	10
Generated Sample Per Class	320	360	380	390
Real Sample for Test Dataset	100	100	100	100

**Table 11:** Comparison of average precision, recall, F1-Score of different methods.

Methods	Precision (%)	Recall (%)	F1-Score (%)
ADASYN+1D	63.2	61.8	62.5
DWGANGP+1D	77.8	76.9	77.3
CBAM-CRLSGAN+1D	79.0	77.7	78.4
AWLT-GAN+1D	80.3	79.2	79.7
ST-CatGAN+2D	81.0	80.1	80.5
Imp-DCGAN+2D	82.1	81.3	81.7
FTGAN+2D	84.0	83.3	83.6
AWLT-GAN+2D	85.7	85.0	85.3

## Data Availability

The data that support the findings of this study are available upon reasonable request from the authors.
